# Supplementary material for: PIP2 depletion and altered endocytosis caused by expression of Alzheimer's disease‐protective variant PLCγ2 R522
Source: EMBO J. 2021 Jul 13;40(17):e105603. doi: 10.15252/embj.2020105603 (PMC8408593; doi:10.15252/embj.2020105603)
Supplement: Supplementary file 2 — Appendix [file EMBJ-40-e105603-s002.pdf]

Appendix: The Alzheimer's disease protective PLCγ2 R522 hypermorph depletes PIP2 and alters endocytosis.

Emily Maguire [#1], Georgina E. Menzies[#1], Thomas Phillips[#1], Michael Sasner[2], Harriet M. Williams[2], Magdalena A. Czubala[3], Neil Evans[1], Emma L Cope[4], Rebecca Sims[5], Gareth R. Howell[2], Emyr Lloyd-Evans[4], Julie Williams[†1,5], Nicholas D. Allen[†4] and Philip R. Taylor[†*1,3].

Table of Contents

Appendix Figure S1. Related to Figure 2,3. Novel R522 knockin mice and validation of mouse cells and specificity of PLCγ2 in response	2
Appendix Figure S2. Related to Figure 4,5. Inhibitors of endocytosis alter the uptake of particles by M-MOPS and primary mouse microglia	4
Appendix Figure S3. Related to Figure 4,5. Knock down of PLCγ2 decrease uptake of particles by M-MOPS and increased levels of PIP_2 increases uptake of zymosan and Ecoli by M_MOPS	6
Appendix Figure S4. Related to Figure 4,5. Inhibitors of phosphoinositide metabolism alter the uptake of particles by M-MOPS	8
Appendix Figure S5. Related to Figure 4,5. Variable dosage of particles alters rate of uptake of particles by M-MOPS	9
Appendix Figure S6: Representative images of PIP2 staining in mouse macrophages and primary microglia	10
Appendix Figure S7: Representative images of microglia in the cortex and hippocampus	11
Appendix Figure S8. CRISPR-engineered R522 allele in human iPSC cells	12
Appendix Table S1 - Hydrogen Bonding	13
Appendix Table S2 – Wild-type and mutated protein simulation stability outputs	14
Appendix Table S2 - Root mean square deviation (RMSD)	14

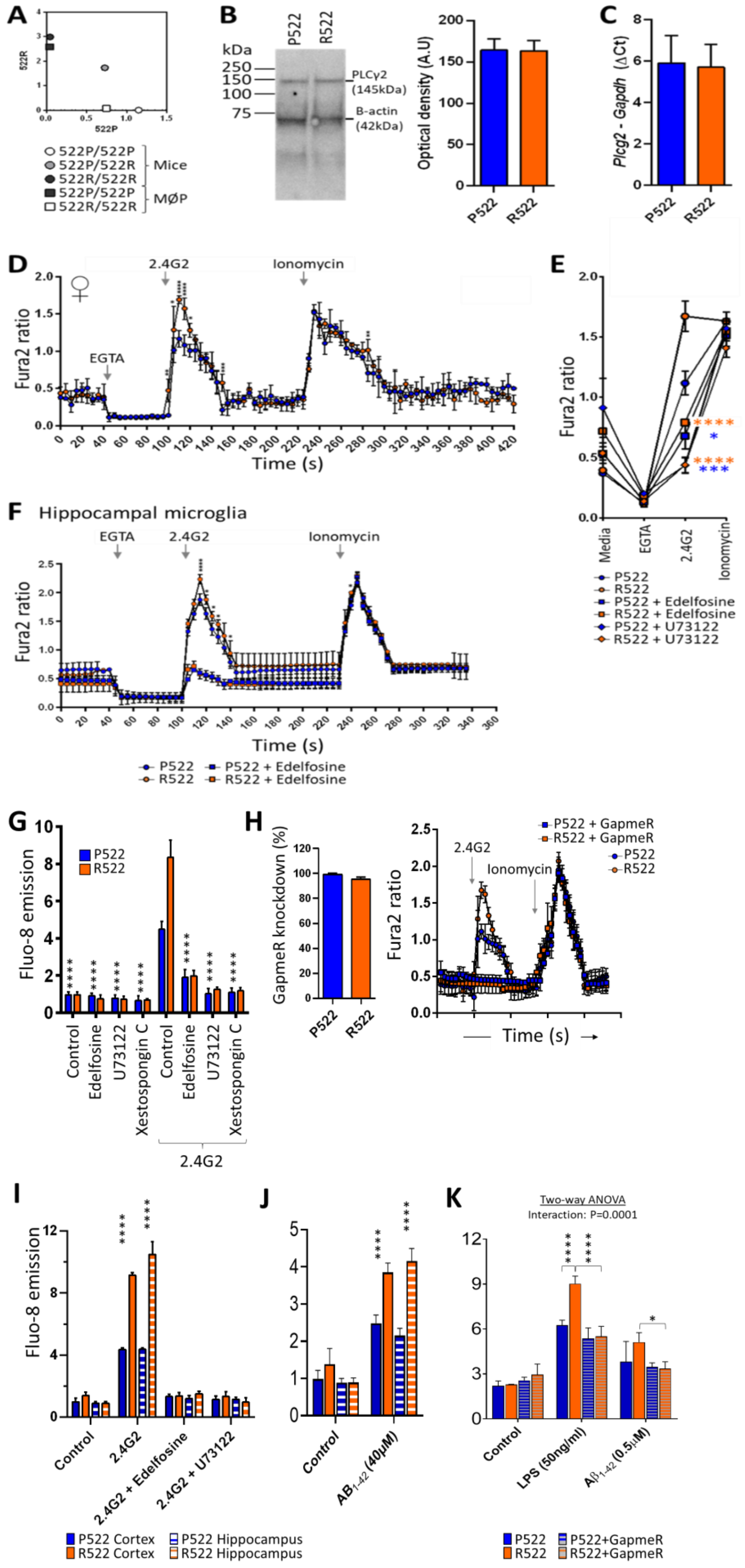

A
522P/522P
522P/522R
522R/522R
522P/522P
522R/522R
Mice
MØP
B
P522
R522
kDa
250
150
100
75
PLCγ2 (145kDa)
B-actin (42kDa)
Optical density (A.U)
C
Plcg2 - Gapdh (ΔCt)
D
2.4G2
Ionomycin
EGTA
Fura2 ratio
Time (s)
E
Media
EGTA
2.4G2
Ionomycin
P522
R522
P522 + Edelfosine
R522 + Edelfosine
P522 + U73122
R522 + U73122
F Hippocampal microglia
G
Fluo-8 emission
Control
Edelfosine
U73122
Xestospongin C
H
GapmeR knockdown (%)
P522 + GapmeR
R522 + GapmeR
I
P522 Cortex
R522 Cortex
P522 Hippocampus
R522 Hippocampus
2.4G2 + Edelfosine
2.4G2 + U73122
J
AB1-42 (40μM)
K
Two-way ANOVA
Interaction: P=0.0001
LPS (50ng/ml)
Aβ1-42 (0.5μM)
P522+GapmeR
R522+GapmeR

Appendix Figure S1. Related to Figure 2,3. Novel R522 knockin mice and validation of mouse cells and specificity of PLCγ2 in response

A. qPCR endpoint analysis of DNA from mice and conditional-immortalised macrophage precursor cells to test for the *Plcg2*^{P522} and *Plcg2*^{R522} genotype. The figure represents which genotype the samples were found to contain. **B.** Western blotting (representative image shown) of PLCγ2 in macrophage precursor cells with the P522 (blue) control and R522 (red) variants. Graph contains data from three repeats optical density normalised to loading control. **C.** qPCR results of gene expression of *Plcg2* in M-MØP cells with the P522 (blue) control and R522 (red) variants. Results normalised to delta change of GADPH housekeeping gene expression. **D.** Fura2 340/380 time traces from M-CSF-differentiated macrophages derived from conditionally-immortalised macrophage precursor cell lines (M-MØP) of macrophages derived from the bone marrow cells of B6 wild type (P522, blue) mice and *Plcg2*^{R522} mice (R522, red). Replicate independent cell lines generated from female mice are shown. Cells were exposed to 5ug/ml anti-FcγRII/III along with EGTA and 2uM Ionomycin as indicated. The data were analysed by two-way ANOVA with Sidak post-tests. **E.** Fura2 340/380 peaks of time traces from M-MØP (blue: P522; red: R522) treated with PLCγ-inhibitors. Cells were exposed to 5µg/ml anti-FcγRII/III along with EGTA and 2µM Ionomycin with or without pre-exposure for 2 hours with Edelfosine (10µM) or U73122 (5µM). Data were analysed by two-way ANOVA with Tukey's multiple comparison test. All graphical data represents mean±SD of 3 independent experiments, except D, where n = 4. **F.** Fura2 340/380 time traces from primary microglia derived from the hippocampus of neonate mice. Cells from B6 wild type (blue: P522) mice and *Plcg2*^{R522} mice (red: R522) with or without pre-exposure for 2 hours with Edelfosine (10µM). Cells were exposed to 5ug/ml anti-FcγRII/III along with EGTA and 2uM Ionomycin. The data were analysed by two-way ANOVA with Sidak's post-tests. Data represents mean±SD of 3 independent experiments. **G.** M-MØP (blue: P522; red: R522) were loaded with Fluo-8 Ca^{2+} indicator and examined for peak changes in fluoresce after exposure to 5ug/ml anti-FcγRII/III (2.4G2) with or without pre-exposure for 2 hours with Edelfosine (10µM) or U73122 (5µM) or Xestospogin C (5µM). **H.** Left panel shows LNA GapmeR percentage knockdown of *Plcg2* gene expression (RNA) in M-MOP cells within the P522 control (blue) and R522 (red) variant lines. Right panel shows Fura2 340/380 time traces from M-MØP cell lines (blue: P522; red: R522) which have undergone *Plcg2* knockdown (square symbols) using an antisense LNA GapmeR. Cells were exposed to 5µg/ml anti-FcγRII/III and 2µM Ionomycin. **I.** Microglia from *Plcg2*^{P522} (blue: P522) mice and *Plcg2*^{R522} mice (red: R522) from neonate cortex (solid colour) or hippocampus (striped) were loaded with Fluo-8 Ca^{2+} indicator. These cells were then examined for peak changes in fluoresce after exposure to 5µg/ml anti-FcγRII/III. These readings were taken with or without pre-exposure for 2 hours with Edelfosine (10µM) or U73122 (5µM). **J** Microglia from *Plcg2*^{P522} (blue: P522) mice and *Plcg2*^{R522} mice (red: R522) from neonate cortex (solid colour) or hippocampus (striped) were loaded with Fluo-8 Ca^{2+} indicator. These cells were then examined for peak changes in fluoresce after exposure to $A\beta_{1-42}$ oligomers (40µM). **K** M-MØP (blue: P522; red: R522) with (stripped) or without (solid colour) LNA GapmeR knockdown of PLCg2 were loaded with Fluo-8 Ca^{2+} indicator. These cells were then examined for peak changes in fluoresce after exposure to LPS (50ng/ml) or $A\beta_{1-42}$ oligomers (40µM). The data were analysed by two-way ANOVA with Sidak's post-tests.. *=p<0.05, **=p<0.01, ***=p<0.001.

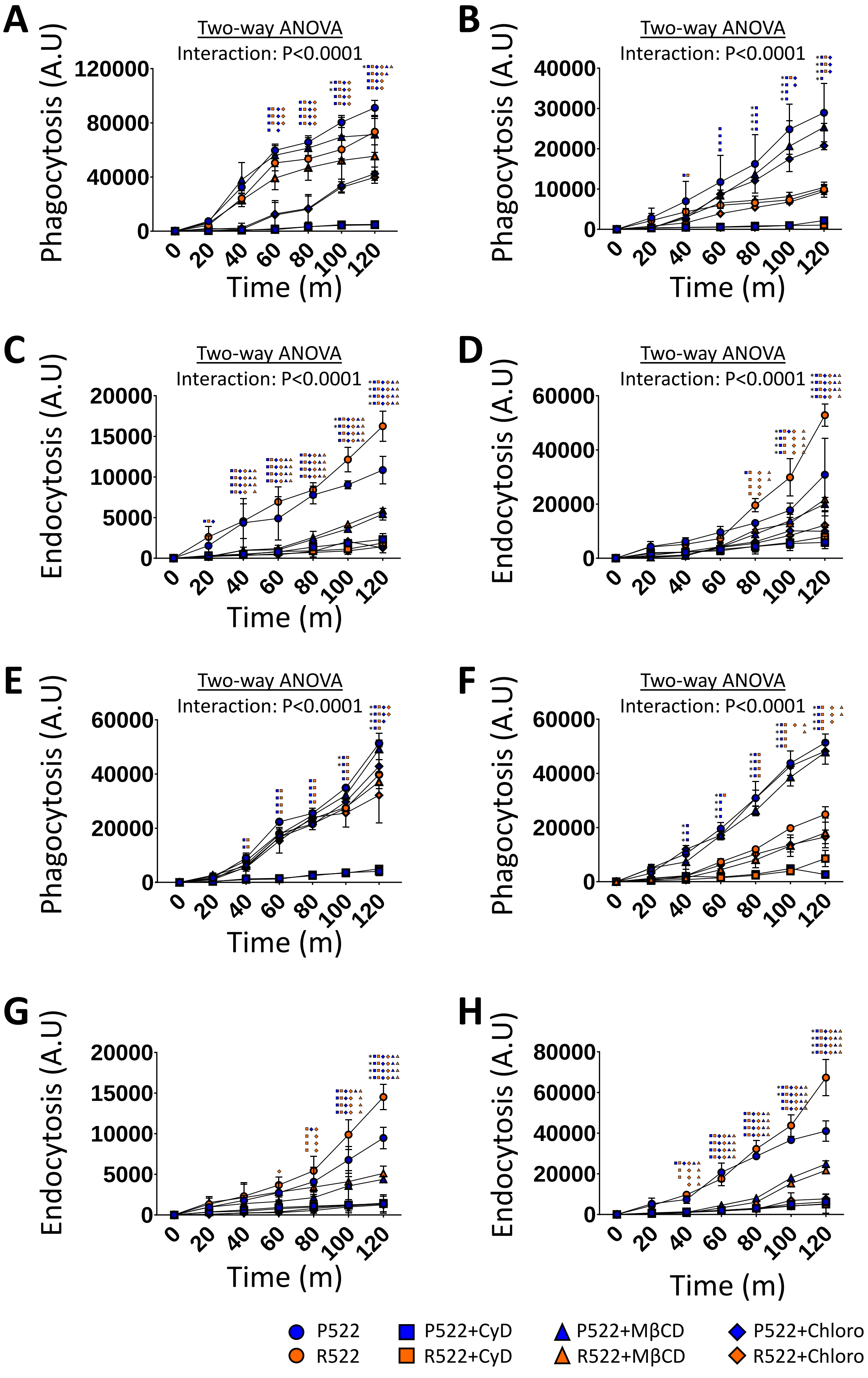
A
Two-way ANOVA
Interaction: P<0.0001
Phagocytosis (A.U)
Time (m)
B
Two-way ANOVA
Interaction: P<0.0001
Phagocytosis (A.U)
Time (m)
C
Two-way ANOVA
Interaction: P<0.0001
Endocytosis (A.U)
Time (m)
D
Two-way ANOVA
Interaction: P<0.0001
Endocytosis (A.U)
Time (m)
E
Two-way ANOVA
Interaction: P<0.0001
Phagocytosis (A.U)
Time (m)
F
Two-way ANOVA
Interaction: P<0.0001
Phagocytosis (A.U)
Time (m)
G
Endocytosis (A.U)
Time (m)
H
Endocytosis (A.U)
Time (m)
P522
R522
P522+CyD
R522+CyD
P522+MβCD
R522+MβCD
P522+Chloro
R522+Chloro

Appendix Figure S2. Related to Figure 4,5. Inhibitors of endocytosis alter the uptake of particles by M-MOPS and primary mouse microglia. Uptake activity of R522 and P522 M-MOP **(A-D)** and primary mouse microglia **(E_H)** was assessed using pHrodo red labelled BioParticles and FITC soluble A$\beta_{1\text{-}42}$ oligomers. Cells were treated with pHrodo red labelled E.coli (A,E), zymosan (B,F), dextran (MW 10000) (C,G) or FITC soluble A$\beta_{1\text{-}42}$ oligomers (D,H). Cells were treated with Cytochalasin D (square), Methyl-β-cyclodextrin (triangle) or chlorpromazine (diamond). All data shows the mean±SD of 3 independent experiments were analysed by two-way ANOVA using Sidak's multiple comparison test. *=p<0.05, **=p<0.01, ***=p<0.001 (blue: P522; red: R522).

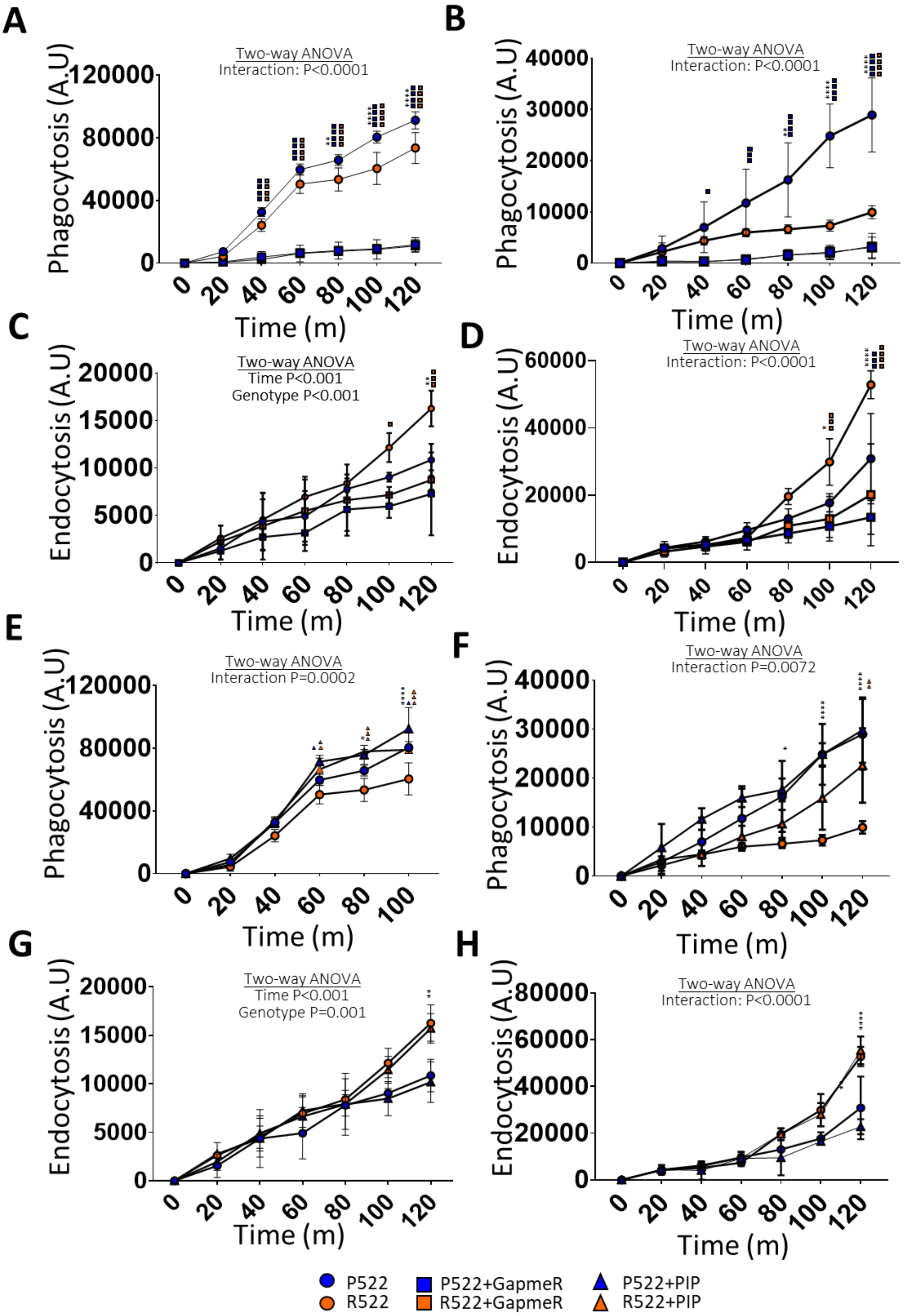

Appendix Figure S3. Related to Figure 4,5. Knock down of PLCγ2 decrease uptake of particles by M-MOPS and increased levels of PIP2 increases uptake of zymosan and Ecoli by M_MOPS. Uptake activity of R522 and P522 M-MOP was assessed using pHrodo red labelled BioParticles and FITC soluble Aβ_{1-42} oligomers. **A-D** M-MØP (blue: P522; red: R522) with (circle) or without (square) LNA GapmeR knockdown of PLCg2 were treated

with phrodo red labelled E.coli (A), zymosan (B), dextran (MW 10000) (C) or FITC soluble Aβ_{1-42} oligomers (D). **E-H** M-MØP (blue: P522; red: R522) with (circle) or without (square) hyper levels of PIP_2 were treated with phrodo red labelled E.coli (E), zymosan (F), dextran (MW 10000) (G) or FITC soluble Aβ_{1-42} oligomers (H). All data shows the mean±SD of 3 independent experiments were analysed by two-way ANOVA using Sidak's multiple comparison test. *=p<0.05, **=p<0.01, ***=p<0.001 (blue: P522; red: R522).

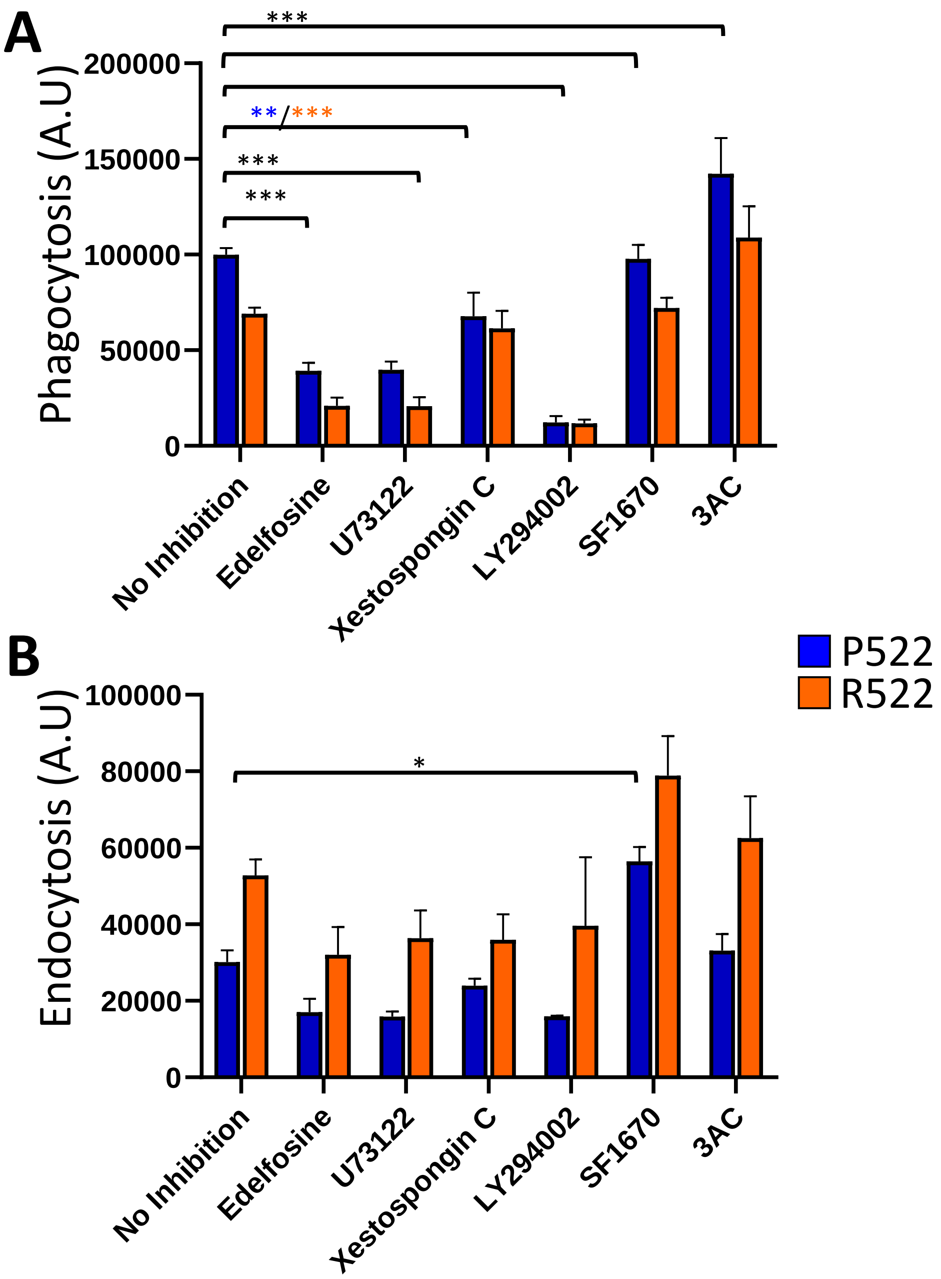

Appendix Figure S4. Related to Figure 4,5. Inhibitors of phosphoinositide metabolism alter the uptake of particles by M-MOPS. Uptake activity of R522 and P522 M-MOP was assessed using pHrodo red labelled E.Coli **(A)** and dextran (MW 10000) **(B).** Arbitrary units (A.U) represent detected fluorescence after two hours. Cells were treated with or without Edelfosine, U73122 (Plcg2 inhibitors), Xestospongin C (IP_3 inhibitor), 3-a-aminocholestane (SHIP 1 inhibitor), SF1670 (PTEN inhibitor) or LY294002 (PI-3K inhibitor). All data shows the mean±SD of 3 independent experiments were analysed by two-way ANOVA using Sidak's multiple comparison test. *=p<0.05, **=p<0.01, ***=p<0.001. (blue: P522; red: R522)

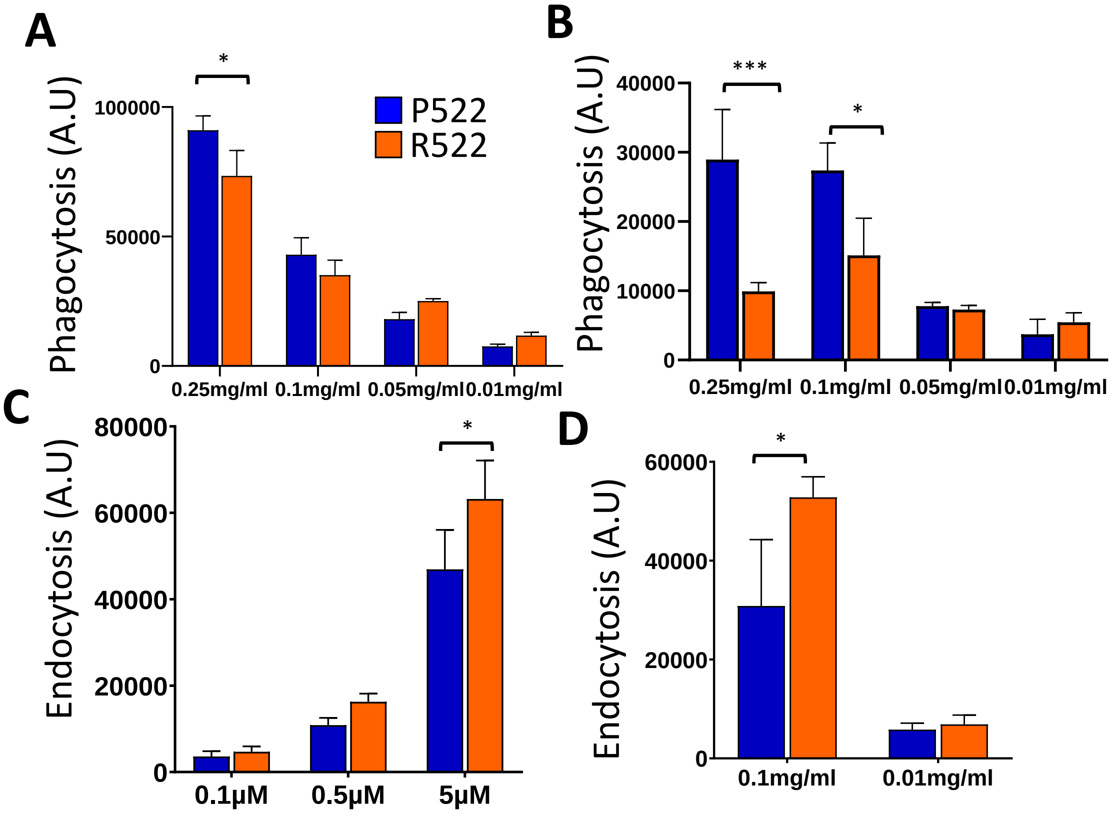

Appendix Figure S5. Related to Figure 4,5. Variable dosage of particles alters rate of uptake of particles by M-MOPS. Uptake activity of R522 and P522 M-MOP was assessed using pHrodo red labelled BioParticles and FITC soluble A$\beta_{1\text{-}42}$ oligomers. Cells were treated with phrodo red labelled E.coli (A), zymosan (B), dextran (MW 10000) (C) or FITC soluble A$\beta_{1\text{-}42}$ oligomers (D). Arbitrary units (A.U) represent detected fluorescence after two hours. All data shows the mean±SD of 3 independent experiments were analysed by two-way ANOVA using Sidak's multiple comparison test. *=p<0.05, **=p<0.01, ***=p<0.001. (blue: P522; red: R522)

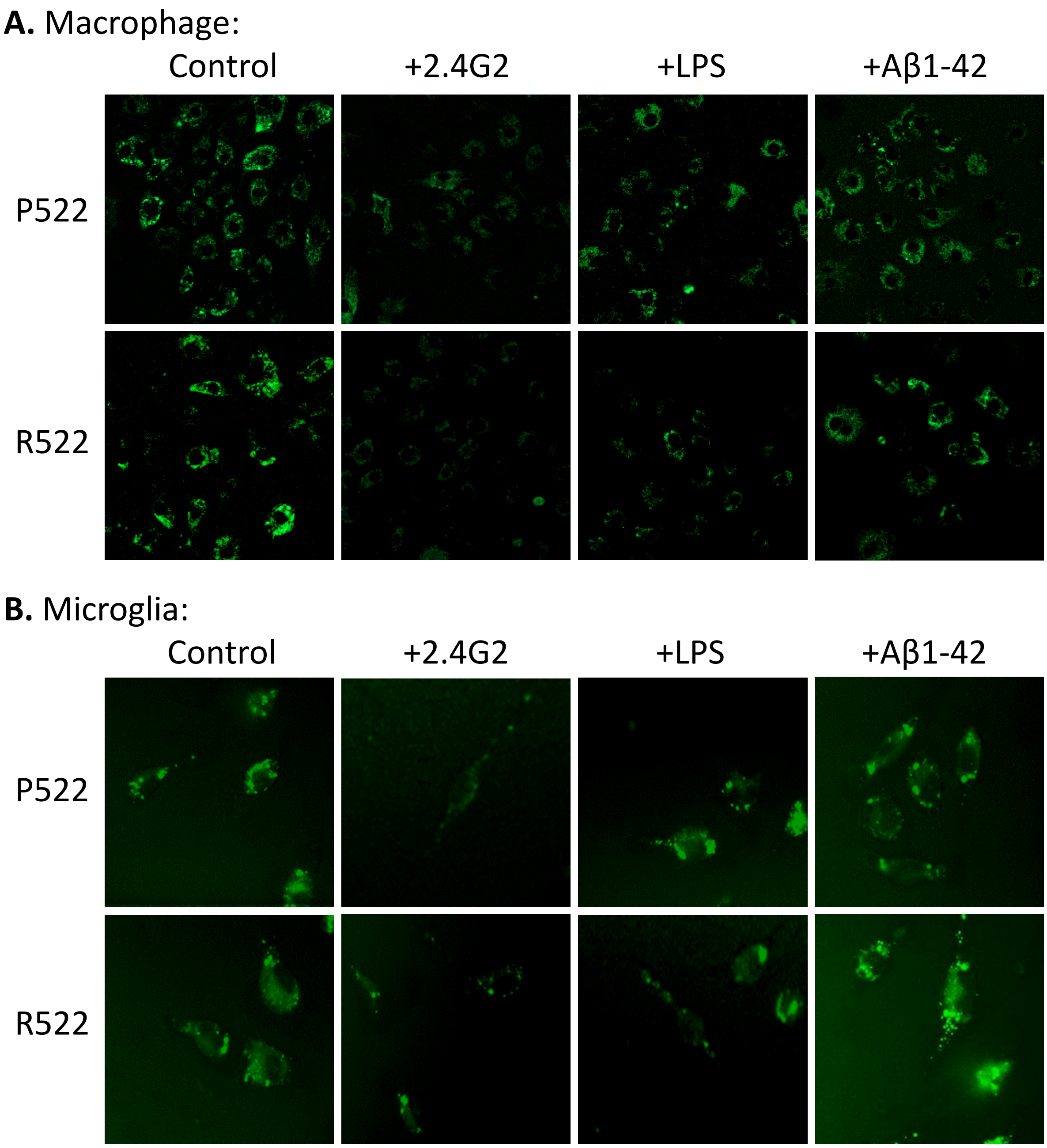

Appendix Figure S6: Representative images of PIP2 staining in mouse macrophages and primary microglia. Images taken of mouse macrophages (A) and primary mouse microglia (B) from the *Plcg2*^{P522} and *Plcg2*^{R522} cell line stained against PIP2 (green). Cells were treated (left to right) with PBS, 2.4G2, $A\beta_{1-42}$ or LPS.

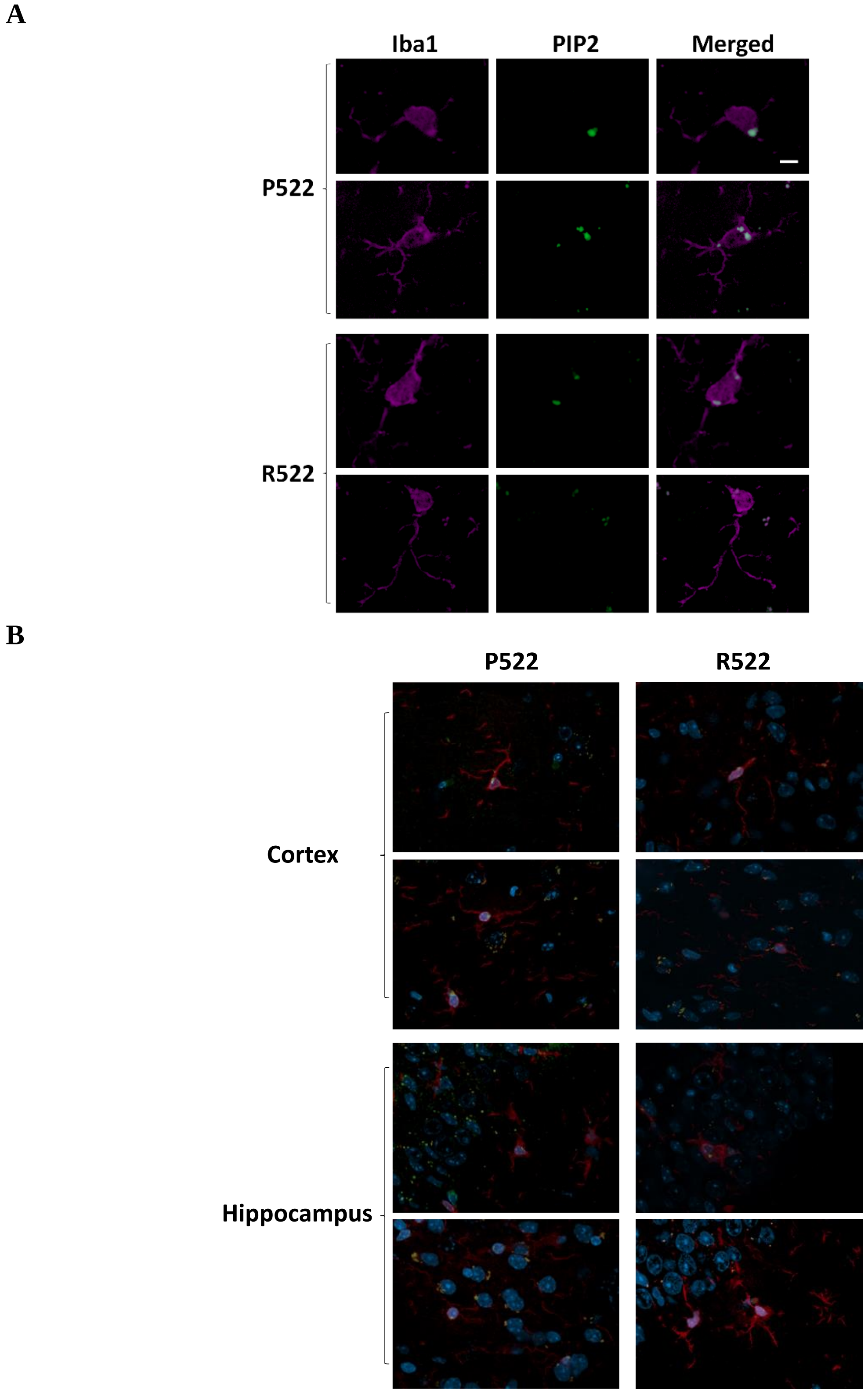

Appendix Figure S7: Representative images of microglia in the cortex and hippocampus. A. Cryosections of *Plcg2*^{R522} and *Plcg2*^{P522} mouse brains were immunostained for Iba1 and PIP2 and images were taken in the cortex. Representative images of the cortex are displayed from *Plcg2*^{P522} and *Plcg2*^{R522} Iba1=Red and PIP_2=Green, scale bar=5µm. **B.** Additional images taken in the cortex (top) and hippocampus (bottom) of *Plcg2*^{P522} (Left) and *Plcg2*^{R522} (Right) mice labelled with Iba1 (red), PIP2 (green) and DAPI (blue).

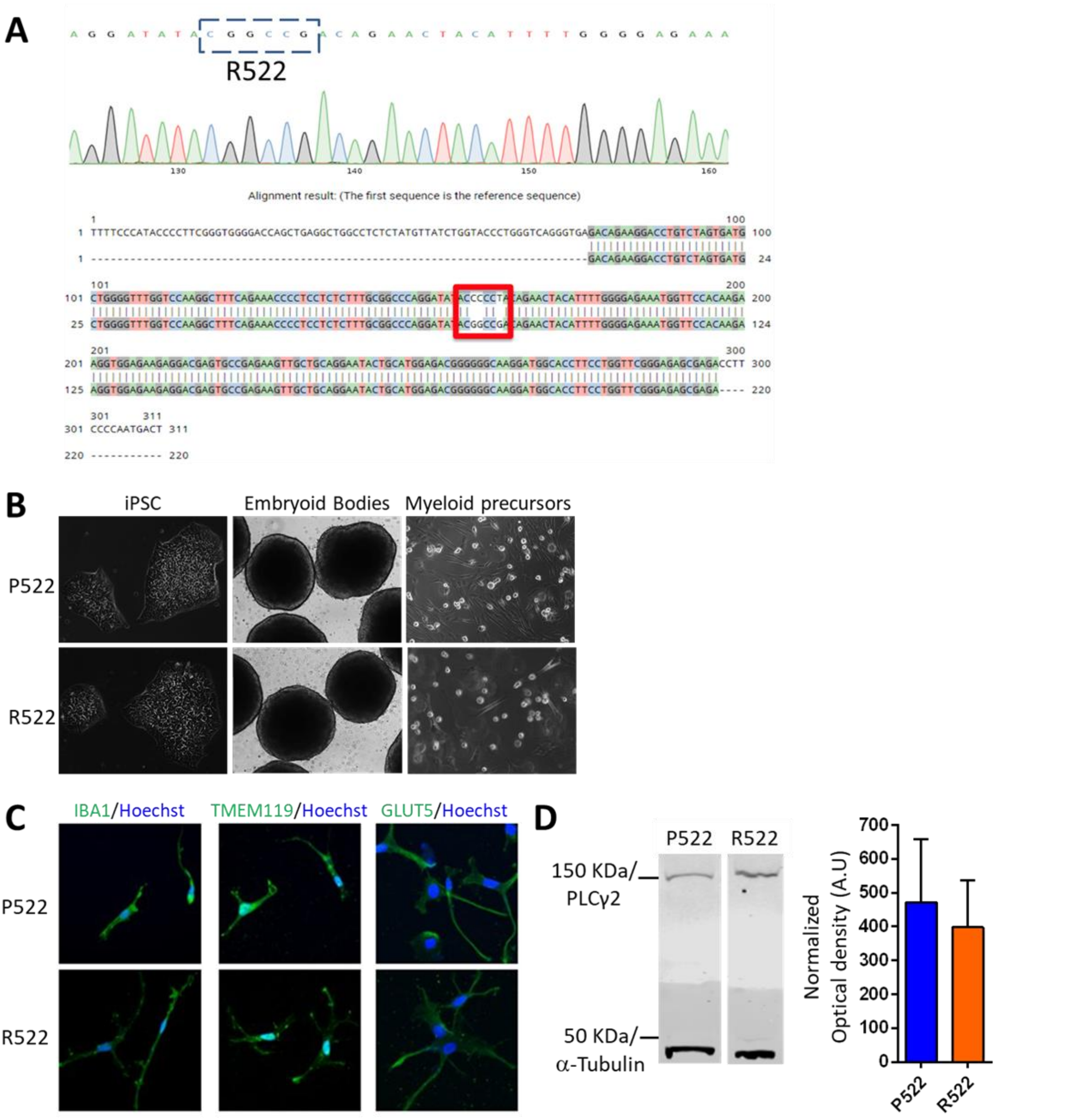

Appendix Figure S8. CRISPR-engineered R522 allele in human iPSC cells.
Isogenic *PLCG2*^{R522} variant iPSC lines were generated using CRISPR-assisted gene editing. Guide-RNAs were combined with Cas9 (HiFi Cas9 nuclease V3) to induce a double stranded break (DSB) in the specific P522 site in *PLCG2*. A P522R oligo was also introduced as a template for DNA repair enzymes. This resulted in the introduction of the mutation and the generation of 9 homozygotes and 7 heterozygotes clones for the R522 variant mutation within the KOLF2 iPSCs. Sequencing confirmed introduction of the P522R mutation (Example shown). **B.** Differentiation of P522R variant iPSCs first to embryoid bodies (EB's) then to myeloid precursors closely mimics the differentiation of control iPSCs. **C.** Immunofluorescent staining indicates a microglial-like phenotype with cells of both lines expressing microglial markers (IBA1, TMEM119 and GLUT5). **D.** Western blotting (representative images shown, left) of PLCγ2 in myeloid precursor cells with the P522 (blue) control and R522 (red) variants quantified (right). Graph contains data from three repeats optical density normalised to loading control, data represents mean±SD.

Appendix Table S3 - Hydrogen Bonding

**WT/522R**	**Donor**	**Acceptor**
WT	T524	P522<br>(two sites)
R522	V517<br>(two sites)	R522
R522	Q519<br>(two sites)	R522
R522	D520	R522
R522	T524<br>(two sites)	R522
R522	R522	Q489<br>(two sites)
R522	R522	E514<br>(two sites)
R522	R522	E515<br>(nine sites)
R522	R522	E516<br>(five sites)
R522	R522	V517<br>(three sites)
R522	R522	P518<br>(four sites)
R522	R522	Q519<br>(eight sites)
R522	R522	D520<br>(nine sites)
R522	R522	I521<br>(three sites)
R522	R522	P523<br>(three sites)
R522	R522	T524<br>(five sites)
R522	R522	E525<br>(nine sites)
R522	R522	E530<br>(five sites)
R522	R522	Y611<br>(three sites)
R522	R522	R618
R522	R522	Q615<br>(five sites)
R522	R522	E721<br>(four sites)

Appendix Table S2 – Wild-type and mutated protein simulation stability outputs

Energy	Average	Standard Err.	Units.
	Wild-type Simulation		
Total Energy	-2.54491e+06	90	(kJ/mol)
Pressure	1.054	0.061	(bar)
Volume	2607.84	0.028	(nm^3)
	Mutated Simulation		
Total Energy	-2.54557e+06	95	(kJ/mol)
Pressure	1.01289	0.014	(bar)
Volume	2607.87	0.016	(nm^3)

Appendix Table S4 - Root mean square deviation (RMSD)

	Wild Type	R522 Mutation
Mean	0.53	0.53
Standard Deviation	0.05	0.09
