# Supplementary figures and images for: PIP2 depletion and altered endocytosis caused by expression of Alzheimer's disease‐protective variant PLCγ2 R522
Source: EMBO J. 2021 Jul 13;40(17):e105603. doi: 10.15252/embj.2020105603 (PMC8408593; doi:10.15252/embj.2020105603)

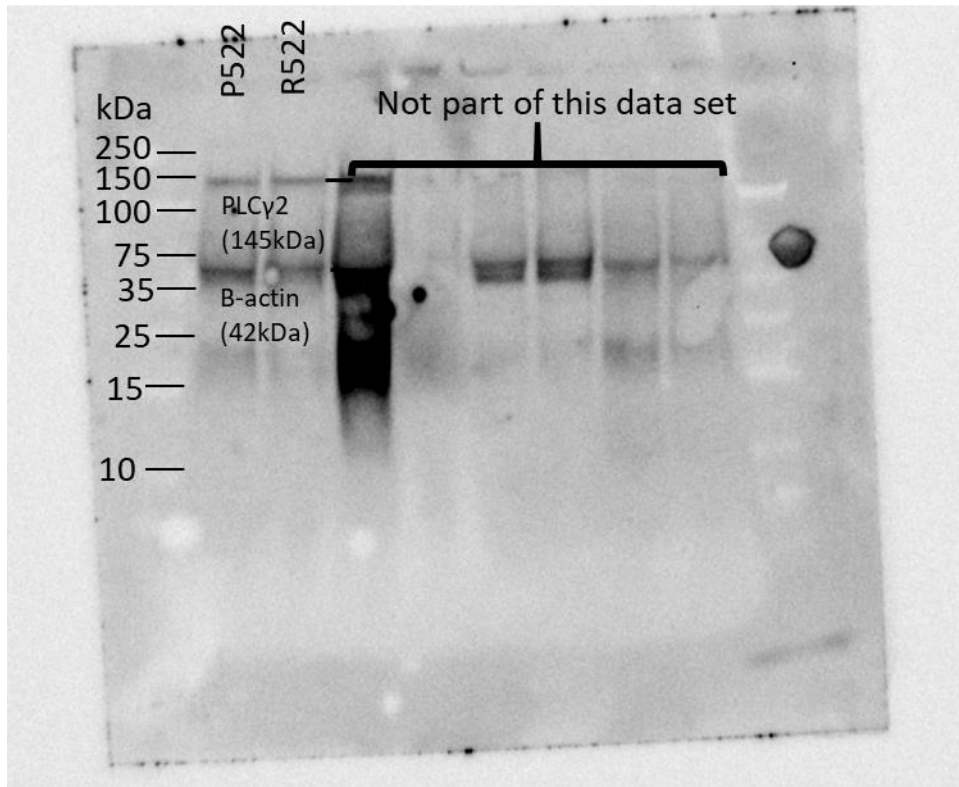

Western blot from Appendix figure S1B

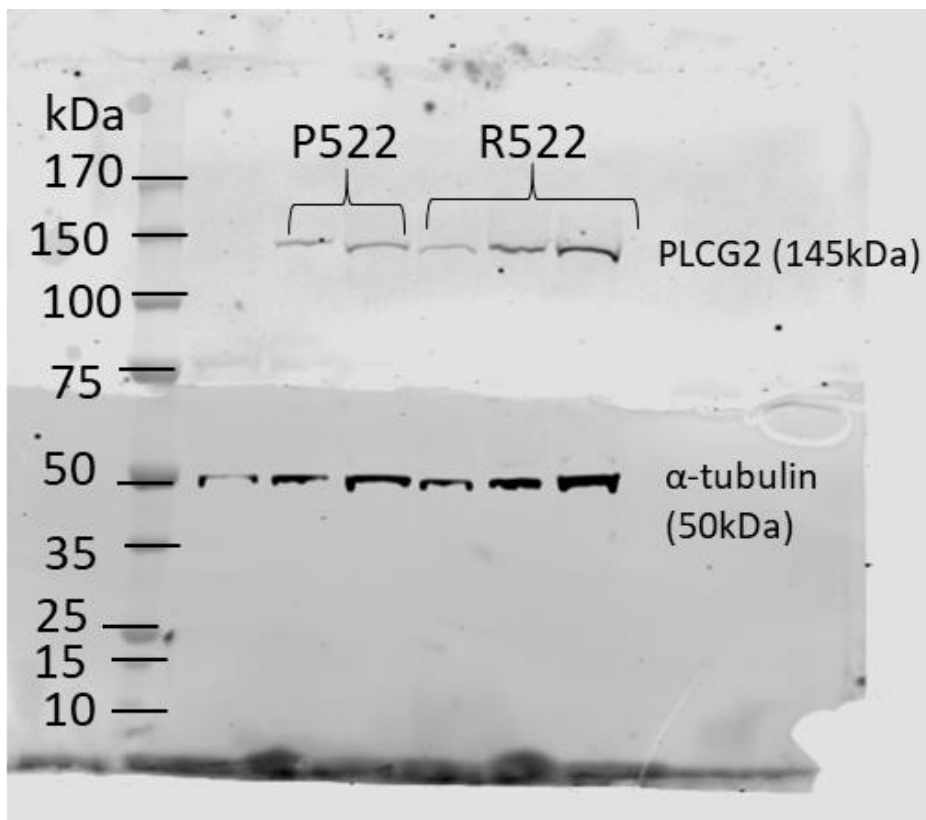

Western blot from Appendix figure S8D

Supplement: Supplementary file 3 — Source Data for Appendix [file EMBJ-40-e105603-s003.pdf]
